# Routine Vaccinations and Reduced Risk of Alzheimer's Disease: A Meta‐Analysis of Epidemiological Evidence (2010–2023)

**DOI:** 10.1002/alz70858_103997

**Published:** 2025-12-25

**Authors:** Padmanayakege C Rupasinghe, Nadir G. Abdelrahman, Faegheh Miryousefiata, Shahd Ahmed, Farjahan Chowdhury

**Affiliations:** ^1^ Icahn School of Medicine at Mount Sinai, New York, NY, USA; ^2^ Michigan State University, East Lansing, MI, USA; ^3^ Samuel merritt university, Oakland, CA, USA

## Abstract

**Background:**

Observational studies suggest routine vaccinations may reduce Alzheimer's disease (AD) risk by modulating immune pathways (Lehrer & Rheinstein, 2020). This study synthesizes epidemiological evidence through meta‐analysis to evaluate the association between vaccination history and AD incidence in adults aged 50 years and older.

**Method:**

A systematic literature search was conducted across PubMed, Cochrane Library, Embase, and Web of Science from 2010–2023. Observational studies with quantifiable effect sizes evaluating vaccination and AD incidence were included. Of 22 eligible studies, 15 met inclusion criteria for meta‐analysis. Data were pooled using random‐effects models (RevMan 5.4, STATA 17). Heterogeneity was assessed using the I^2^ statistic, and publication bias was evaluated using Egger's test. Mechanistic pathways were illustrated based on existing literature.

**Result:**

Meta‐analysis of 15 studies comprising 12 million participants revealed significant associations between routine vaccinations and reduced AD risk. Influenza vaccination was associated with a pooled hazard ratio (HR) of 0.62 (95% CI: 0.57–0.68, *p* <0.001; I^2^=65%). Pneumococcal vaccination demonstrated a pooled odds ratio (OR) of 0.71 (95% CI: 0.63–0.80, *p* <0.001; I^2^=58%), while tetanus‐diphtheria vaccination showed a pooled OR of 0.51 (95% CI: 0.44–0.60, *p* <0.001; I^2^=42%). Egger's test indicated no significant publication bias (*p* = 0.15). Mechanistic insights highlighted reduced systemic inflammation, enhanced microglial activation, and amyloid‐beta clearance as potential neuroprotective pathways.

**Conclusion:**

Routine vaccinations are associated with a 27–49% lower risk of AD, supporting immune modulation as a plausible neuroprotective mechanism. While these observational findings preclude causal inference, they emphasize vaccinations’ potential role in dementia prevention. Randomized trials are necessary to validate these associations and inform public health strategies.

